# Extracellular volume MRI increases the detection of myocardial abnormalities beyond late gadolinium enhancement - initial findings

**DOI:** 10.1186/1532-429X-14-S1-P291

**Published:** 2012-02-01

**Authors:** Magnus Lundin, Peder Sorensson, Anders Gabrielsen, Peter Kellman, Martin Ugander

**Affiliations:** 1 Department of Clinical Physiology, Karolinska Institute, Stockholm, Sweden; 2 Department of Cardiology, Karolinska Institute, Stockholm, Sweden; 3 National Heart, Lung and Blood Institute, National Institutes of Health, Bethesda, MD, USA

## Background

Late gadolinium enhancement (LGE) is excellent for identifying focal lesions in the myocardium (necrosis, fibrosis), but diffuse homogeneous abnormalities are not readily detectable. Quantitative measurement of the myocardial extracellular volume (ECV) fraction using MRI has recently been validated histologically, and implemented for parametric imaging in patients. We hypothesized that quantification of ECV using ECV imaging could detect focal lesions detected by LGE, and diffuse homogenous disturbances in the myocardial extracellular volume fraction which are not detectable by LGE. The objective of the study was to examine the diagnostic utility of ECV MRI for detecting myocardial tissue abnormalities compared to LGE.

## Methods

Consecutive patients referred for clinical cardiac MRI evaluation of known or suspected heart disease were prospectively enrolled (n=32, mean age 51 years, range 13-73, 63% male). MRI was undertaken at 1.5T using a Modified Look-Locker Inversion recovery (MOLLI) sequence before and 15 minutes after an intravenous bolus of a gadolinium-based extracellular contrast agent (gadoteric acid, 0.2 mmol/kg). Maps of the extracellular volume fraction (ECV images) were generated using MOLLI-derived T1-mapping before and after contrast, calibrated to hematocrit from a venous blood sample. Motion correction and co-registration was performed offline using an automated dedicated algorithm. ECV images were interpreted quantitatively and the results were compared to clinical assessment of LGE images. Increased myocardial ECV was determined by quantitative comparison to established normal values where the 95% confidence limits are 20-32%.

## Results

Out of 32 patients, 10 had focal lesions (myocardial infarction, n=6, lesion ECV range 38-72%, or non-ischemic lesions, n=4, lesion ECV range 38-74%). All lesions identified by increased ECV were also identified by blinded clinical read of LGE images (100% agreement). Among patients with normal LGE findings (n=22), two patients (9%) had diffusely increased myocardial ECV (dilated cardiomyopathy, mean ECV 40%, and hypertrophic cardiomyopathy, mean ECV 40%). The figure shows examples of LGE and ECV images in a normal finding, a focal lesion and a case with diffusely increased ECV.

**Figure 1:**
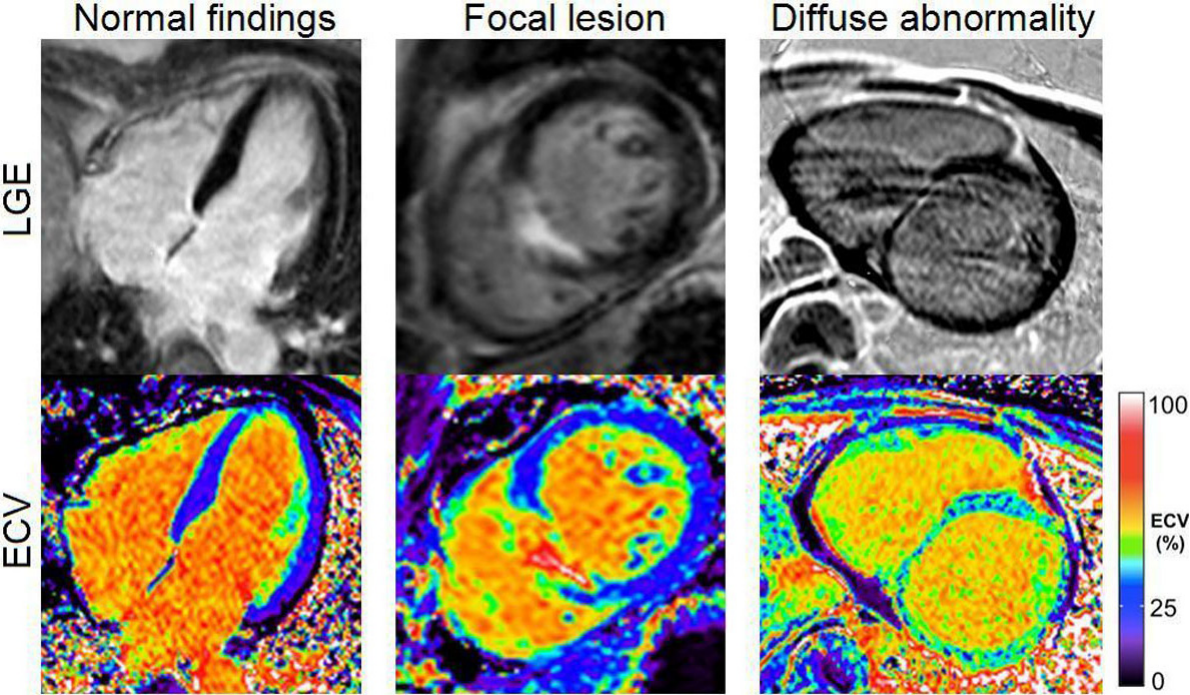
Late gadolinium enhancement (LGE) images (top row) and extracellular volume (ECV) images (bottom row). Images from three different patients illustrating normal findings (left), a focal lesion following myocardial infarction (center), and a diffuse abnormality due to dilated cardiomyopathy (right). The color bar shows the proportion of tissue comprised of extracellular volume.

## Conclusions

Our initial findings suggest that ECV and LGE imaging have excellent diagnostic agreement for identifying focal myocardial findings such as those seen in myocardial infarction and non-ischemic heart disease. The ability of ECV imaging to quantify and detect diffuse abnormalities in myocardial ECV increased the number of diagnostic findings compared to LGE in 9% of cases with otherwise normal LGE. Further studies in larger populations are warranted to explore the diagnostic and prognostic benefits of ECV imaging in assessment of diffuse pathologies of the myocardial extracellular space.

## Funding

The study has been funded in part by a grant (PI: Dr. Ugander) from the Swedish Society for Medical Research.

